# Collet–Sicard syndrome resulting from a Skull Base Paraganglioma: a case report

**DOI:** 10.1093/omcr/omag074

**Published:** 2026-05-24

**Authors:** Abhishek Man Shrestha, Subodh Bashyal, Suman Bhattarai, Lekhjung Thapa

**Affiliations:** Department of Neurology, National Neuro Center, Maharajgunj, Kathmandu 44600, Nepal; Department of Internal Medicine, Universal College of Medical Sciences, Tribhuvan University, Bhairahawa 32900, Nepal; Department of Neurology, National Neuro Center, Maharajgunj, Kathmandu 44600, Nepal; Department of Neurology, National Neuro Center, Maharajgunj, Kathmandu 44600, Nepal

**Keywords:** Collet Sicard syndrome, skull base paraganglioma, cranial nerve palsy, jugular foramen, hypoglossal canal

## Abstract

Collet–Sicard syndrome is a rare neurological condition resulting from lesions at the jugular foramen and hypoglossal canal, leading to combined palsy of cranial nerves IX, X, XI, and XII. Neoplastic causes are most commonly involved, while benign tumors such as paragangliomas are infrequent etiologies. We report a 58-year-old woman who presented with progressive dysphagia and unilateral lower cranial nerve dysfunction. Clinical examination demonstrated right-sided palsy of cranial nerves IX, X, XI, and XII. Magnetic resonance imaging revealed a lobulated, avidly enhancing lesion centered in the right hypoglossal canal with extension into the jugular foramen and adjacent carotid space, radiologically suggestive of a skull base paraganglioma. Based on the characteristic clinical and radiological findings, a diagnosis of Collet–Sicard syndrome secondary to a skull-base paraganglioma was made. This case underscores the importance of through neurological examination and early targeted imaging in patients presenting with progressive dysphagia and unilateral lower cranial nerve palsies.

## Introduction

Collet–Sicard syndrome (CSS), also known as occipital condyle–jugular foramen junction syndrome, is a very rare neurological disorder resulting from a lesion affecting the jugular foramen and hypoglossal canal. This leads to peripheral paralysis of four cranial nerves; the glossopharyngeal (IX), vagus (X), accessory (XI), and hypoglossal (XII) nerves [1]. First described by Collet and Sicard in the early 20th century, the syndrome represents a distinct clinico-anatomical entity owing to the close anatomical proximity of these neurovascular structures at the occipital condyle–jugular foramen junction [2, 3]. Clinically, CSS manifests with dysphagia, dysphonia, hoarseness of voice, ipsilateral palatal weakness, shoulder girdle weakness, and tongue deviation with atrophy, often leading to significant morbidity [4].

The etiological spectrum of CSS is broad and includes traumatic, vascular, inflammatory, infectious, and neoplastic causes. Among these, tumors constitute the most common underlying pathology, accounting for a substantial proportion of reported cases, with metastatic malignancies to the skull base being the predominant cause [5]. Primary benign intracranial tumors are comparatively uncommon etiologies. Paragangliomas, arising from paraganglionic tissue associated with cranial nerves and major vessels, are rare. Although paragangliomas frequently present with isolated lower cranial nerve deficits, their presentation as complete CSS remains exceedingly rare [6].

We report a rare case of CSS secondary to a skull-base paraganglioma, highlighting the clinical presentation, radiological features, and diagnostic challenges associated with this uncommon but clinically significant condition.

## Case report

A 58-year-old woman presented with an insidious onset and progressively worsening dysphagia associated with persistent pain localized to the right side of the neck. She had hypothyroidism and was on levothyroxine 62.5 μg once daily, long-standing hypertension for 15 years treated with atenolol 50 mg twice daily, and an anxiety disorder on sertraline 50 mg at bedtime. Neurological and otolaryngological examination revealed features of right sided lower cranial nerve dysfunction, including reduced elevation of the right side of the soft palate with diminished gag reflex suggestive of CN IX palsy, immobility of the right vocal cord consistent with CN X involvement, weakness of right shoulder elevation with impaired contralateral head rotation due to sternocleidomastoid weakness indicating spinal CN XI palsy, and deviation of the tongue to the right with ipsilateral atrophy and fasciculations, confirming CN XII palsy (Table 1). These findings were clinically consistent with CSS.

**Table 1:** Cranial nerve deficits and clinical correlates.

Clinical examination finding	Clinical correlates
Reduced palatal movement on the right side	Right 9th cranial nerve palsy
Diminished gag reflex on the right side Reduced movement of the right vocal cord.	Right 10th cranial nerve palsy
Weak right shoulder elevation and weak lateral head turn to the left (Sternocleidomastoid).	Right 11th cranial nerve palsy
Deviation of the tongue to the right side with ipsilateral atrophy and fasciculations.	Right 12th cranial nerve palsy

On inspection of the oral cavity during tongue protrusion revealed deviation of the tongue toward the right side, accompanied by visible ipsilateral atrophy and fasciculations of the tongue musculature, consistent with a CN XII palsy. Nasopharyngolaryngoscopic evaluation demonstrated fixation of the right vocal cord with a mild phonatory gap, confirming concomitant involvement of the CN X (Fig. 2).

**Figure 1:**
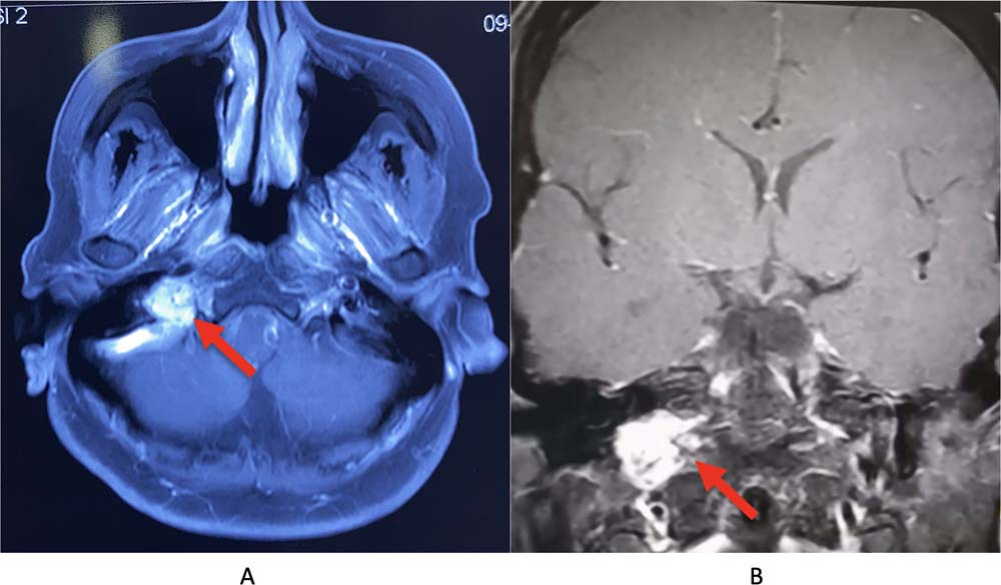
Lobulated enhancing mass centered in the right hypoglossal canal extending to the jugular foramen and carotid space showing T1 iso- to hypointense and T2/FLAIR hyperintense without diffusion restriction with extensions as described- likely paraganglioma (A: Axial view, B: Coronal view).

Magnetic resonance imaging (MRI) of the brain with contrast demonstrated a lobulated, avidly enhancing soft tissue mass centered in the right hypoglossal canal with extension into the right jugular foramen and adjacent carotid space (Fig. 1A and B). The lesion appeared iso- to hypointense on T1-weighted sequences and hyperintense on T2/FLAIR images, without evidence of diffusion restriction. Based on these imaging characteristics, a diagnosis of skull base paraganglioma was made, considering schwannoma as a differential diagnosis, and histopathological correlation was advised. Additional radiological findings included features of right vocal cord palsy, correlating with clinical CN X involvement, and significant thickening of the right lateral glossoepiglottic fold with mild post contrast enhancement, for which nasopharyngolaryngoscopic correlation was suggested. Scattered discrete T2/FLAIR hyperintense foci in the bilateral frontal and parietal deep white matter were also noted, consistent with chronic small vessel ischemic changes.

**Figure 2:**
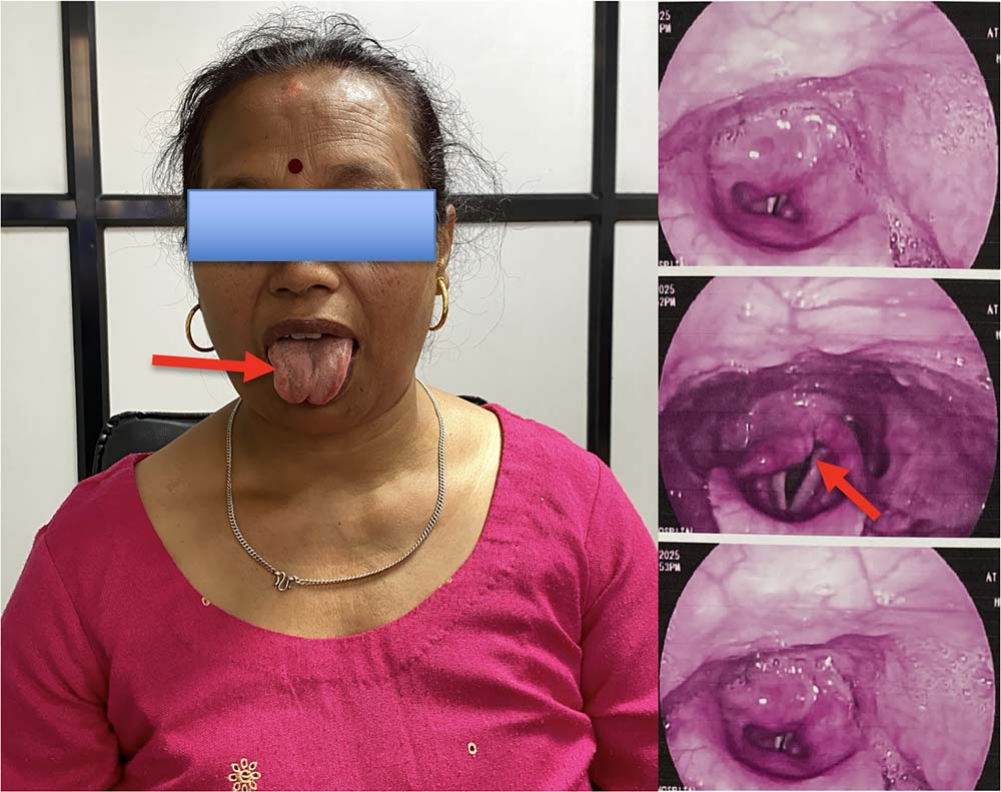
Clinical photograph showing deviation of the tongue to the right side with ipsilateral atrophy of tongue muscles (arrow) consistent with hypoglossal nerve palsy and mild phonatory gap with vocal cord fixed.

In view of the characteristic clinical involvement of cranial nerves IX, X, XI, and XII and radiological evidence of a skull-base lesion involving both the jugular foramen and hypoglossal canal, a diagnosis of CSS secondary to a skull base paraganglioma was established. A diagnostic biopsy was not considered as characteristic imaging findings and clinical presentation were sufficient for diagnosis, and routine biopsy is generally avoided in highly vascular skull-base paragangliomas. Supportive measures such as swallowing rehabilitation, speech and vocal therapy, and strict optimization of blood pressure control were emphasized. Although the tumor control is generally favorable in benign paragangliomas, the patient was counseled regarding the possibility of persistent lower cranial nerve deficits and the ongoing risk of aspiration if dysphagia progresses without timely intervention.

## Discussion

CSS is a rare condition resulting from simultaneous involvement of the jugular foramen and hypoglossal canal, leading to combined dysfunction of cranial nerves IX, X, XI, and XII [1]. The pattern observed in our patient was progressive dysphagia followed by sequential lower cranial nerve palsies. This reflects the anatomical vulnerability of these closely related neurovascular structures and is consistent with descriptions in previously reported cases [5, 7].

Neoplastic lesions remain the most common cause of CSS. However, as some literature stated, our case demonstrates that benign tumors like paragangliomas can also express the full clinical spectrum when there is extension into both the jugular foramen and hypoglossal canal. While paragangliomas are well known to cause isolated lower cranial nerve deficits [6], complete CSS, as seen in our patient has been reported only rarely.

The orderly progression of symptoms in this case strongly suggested a skull-base lesion, a feature that aligns with prior studies emphasizing the importance of clinical pattern recognition. Magnetic resonance imaging was crucial for lesion localization, and the imaging characteristics in our patient were similar with those previously described for skull-base paragangliomas [8, 9]. Although histopathological confirmation was not made, the clinicoradiological correlation was sufficiently characteristic to support the diagnosis.

As reported in previous studies, management requires a multidisciplinary approach, and neurological recovery is often incomplete despite treatment. This case reinforces existing evidence that early recognition and targeted imaging are essential to minimize irreversible lower cranial nerve dysfunction [10].

Thus, this case report highlights the clinical importance of recognizing CSS as a rare but significant cause of progressive dysphagia and unilateral lower cranial nerve palsy. The meticulous neurological examination, systematic clinicoradiological correlation, and appropriate use of magnetic resonance imaging enabled accurate localization of the lesion and identification of an uncommon etiology, namely a skull base paraganglioma. Early and targeted neuroimaging is essential in such presentations to prevent diagnostic delay and irreversible neurological deficits.
